# Reinforcing Low-Volume Fraction Nano-TiN Particulates to Monolithical, Pure Mg for Enhanced Tensile and Compressive Response

**DOI:** 10.3390/ma9030134

**Published:** 2016-02-26

**Authors:** Ganesh Kumar Meenashisundaram, Mui Hoon Nai, Abdulhakim Almajid, Manoj Gupta

**Affiliations:** 1 Department of Mechanical Engineering, National University of Singapore, 9 Engineering Drive 1, Singapore 117576, Singapore; ganesh_kumar@u.nus.edu (G.K.M.); mbinmhb@nus.edu.sg (M.H.N.); 2 Mechanical Engineering Department, College of Engineering, King Saud University, PO Box 800, Riyadh 11421, Saudi Arabia; aalmajid@ksu.edu.sa

**Keywords:** Mg (0.58, 0.97, 1.98, 2.5) vol. % TiN, nanocomposites, grain refinement, X-ray diffraction, tensile, compression, Hall-Petch mechanism, titanium (metal or ceramic), nanoparticulates

## Abstract

Novel Mg (0.58, 0.97, 1.98 and 2.5) vol. % TiN nanocomposites containing titanium nitride (TiN) nanoparticulates of ~20 nm size are successfully synthesized by a disintegrated melt deposition technique followed by hot extrusion. Microstructural characterization of Mg-TiN nanocomposites indicate significant grain refinement with Mg 2.5 vol. % TiN exhibiting a minimum grain size of ~11 μm. X-ray diffraction studies of Mg-TiN nanocomposites indicate that addition of up to 1.98 vol. % TiN nanoparticulates aids in modifying the strong basal texture of pure Mg. An attempt is made to study the effects of the type of titanium (metal or ceramic), size, and volume fraction addition of nanoparticulates on the microstructural and mechanical properties of pure magnesium. Among the major strengthening mechanisms contributing to the strength of Mg-Ti-based nanocomposites, Hall-Petch strengthening was found to play a vital role. The synthesized Mg-TiN nanocomposites exhibited superior tensile and compression properties indicating significant improvement in the fracture strain values of pure magnesium under loading. Under tensile and compression loading the presence of titanium (metal or ductile phase) nanoparticulates were found to contribute more towards the strengthening, whereas ceramics of titanium (brittle phases) contribute more towards the ductility of pure magnesium.

## 1. Introduction

For several years, research and development on lightweight materials has been of particular interest owing to the significant emphasis on greenhouse gas reduction and improving fuel efficiency, especially in the transportation sector. For every 10% of total vehicle weight reduction, vehicle fuel economy has been estimated to be improved by 7% [1]. Magnesium (Mg), with high specific mechanical properties, is a potential candidate material for realizing lightweight construction. With a density of 1.74 g/cm^3^, Mg is the lightest of all the structural metals and has other important properties, such as good castability, high thermal stability, good damping characteristics, and resistance to electromagnetic radiation [2,3,4]. Further, Mg materials have also recently become of great interest for biomedical applications. Low strength, insufficient ductility, and high thermal coefficient of expansion are some of the deficits of Mg materials, which restricts their extensive applications. Simultaneous improvements in the strength and ductility of magnesium materials can be effectively realized by dispersion strengthening for which selection of suitable reinforcements is of prime importance.

Reinforcements can be classified based on their type such as: (a) metallic and (b) ceramic, and size such as (a) micron (1 × 10^−6^ to 1 × 10^−4^), (b) submicron (1 × 10^−7^ to 1 × 10^−6^), and (c) nano (1 × 10^−9^ to 1 × 10^−7^). Among the reinforcement types, more attention on altering the properties of magnesium by addition of inexpensive nanoparticulates (NPs) is highly noticeable. Titanium (Ti) has excellent corrosion resistance and high specific strength. Due to their high cost, Ti and Ti alloys are mostly used in the aerospace industry, racing cars, and special purpose parts for the automobile industry [5]. Further, Ti and Ti alloys are considered as the most attractive metallic materials for biomedical applications targeting permanent implants [6]. They are used for load bearing applications, especially in orthopedics [6].

Ceramics of Ti include titanium carbide (TiC), titanium nitride (TiN), titanium dioxide (TiO_2_), and titanium diboride (TiB_2_). Among the ceramics of Ti, TiC, TiB_2_, and TiN have exceptional hardness, modulus, and have high resistance to erosion and corrosion properties. They are used as coatings for improving the wear resistance of implants. Even though the mechanical properties of TiO_2_ is comparatively lower than the other ceramics of Ti, low cost, non-toxic, chemical and biological stability properties make TiO_2_ an attractive reinforcement for metal matrix composites [7]. Further, TiO_2_ is a bioactive material and a preferred reinforcement to improve the bioactivity of composites targeting biomedical applications [8]. Previously, the authors have studied the effects of low-volume fraction Ti [9] and ceramics of Ti (TiB_2_ [10], TiC [11], and TiO_2_ [12]) on the microstructural and mechanical properties of pure Mg synthesized by utilizing a disintegrated melt deposition technique followed by hot extrusion. The results of the literature search, however, reveal that no attempt is made to date to study the effects of TiN NPs to alter the tensile and compressive response of monolithic, pure Mg in the absence of microstructural factors related to the presence of precipitates and heat treatment.

Accordingly, in the present study, Mg matrix reinforced with low volume fraction (0.58, 0.97, 1.98, and 2.5) vol. % TiN NPs are synthesized by a disintegrated melt deposition technique followed by hot extrusion. The hot extruded Mg-TiN nanocomposite samples were then characterized for physical, microstructural, and mechanical properties and compared to that of previously-synthesized low-volume fraction Ti (metallic) and ceramics of Ti (TiB_2_, TiC and TiO_2_) reinforced Mg nanocomposites. Particular emphasis of this study is to analyze the effects of type of reinforcements (metallic and ceramic Ti NPs) on the microstructural and mechanical properties of pure Mg.

## 2. Results

### 2.1. Density and Porosity Measurements

The results of density measurements of the synthesized pure Mg and Mg-TiN nanocomposites are shown in Table 1. The reference density of the Mg-TiN nanocomposites are theoretically calculated using the rule of mixtures. With the addition of up to 2.5 vol. % TiN NPs, a marginal increase in the experimental density value of pure Mg was observed and Mg 2.5 vol. % TiN nanocomposite exhibited a maximum of ~1.8225 g/cm^3^, which is only ~5% greater than that of pure Mg. Further, the porosity value of the synthesized Mg materials was found to increase with the addition of TiN NPs and Mg 2.5 vol. % TiN nanocomposite exhibited a maximum porosity of ~0.5%.

### 2.2. X-ray Diffraction Studies

**Figure 1:**
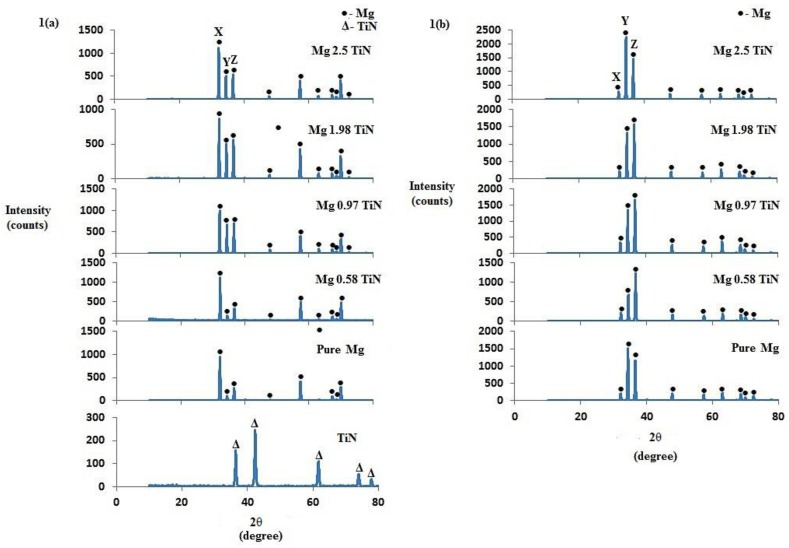
X-ray diffractograms of TiN nanopowder, pure Mg, and Mg-TiN nanocomposites taken along: (**a**) transverse direction and (**b**) longitudinal direction of hot extruded samples. X, Y, and Z represent 2θ = 32°, 34°, and 36° corresponding to (10–10) prism, (0002) basal, and (10–11) pyramidal planes, respectively.

Figure 1 shows the X-Ray diffractogram of TiN and hot extruded pure Mg, Mg-TiN nanocomposites taken along transverse and longitudinal sections of the samples. The high intensity Mg peaks were prominently seen and the peaks corresponding to TiN were not visible in the synthesized Mg-TiN nanocomposites which is due to the limitation of filtered X-rays to detect secondary phases with a low-volume fraction [13]. However, the presence of TiN NPs in the Mg-TiN nanocomposites can be confirmed through microstructural characterization and changes in the intensity of Mg peaks observed through X-ray diffraction studies. In the X-ray diffractogram of as-extruded pure Mg, the peaks observed at 2θ = 32°, 34°, and 36°correspond to prismatic (10–10), basal (0002), and pyramidal (10–11) planes, respectively. With the addition of TiN NPs, changes in the intensity of basal plane of pure Mg were observed. The ratio of XRD intensities of Mg (such as pyramidal, basal, and prismatic) to the maximum XRD intensity (I/I_max_) observed for the synthesized pure Mg and Mg-TiN nanocomposites is given in Table 2.

Along the transverse direction (perpendicular to the extrusion direction), the intensity corresponding to the basal plane of pure Mg was found to increase with up to 0.97 vol. % TiN NPs addition and Mg 0.97 TiN exhibited a maximum I_basal_/I_max_ value of ~0.674. With further addition of TiN NPs, the intensity corresponding to the basal plane of pure Mg was found to decrease and among the synthesized Mg-TiN nanocomposites, Mg 2.5 vol. % TiN exhibited a minimum I_basal_/I_max_ of ~0.460. Further, the intensity corresponding to the prismatic plane (2θ = 32°) was found to be the maximum for all the synthesized Mg materials. Along the longitudinal direction (parallel to the extrusion direction), pure Mg exhibited strong basal texture with maximum XRD intensity corresponding to the basal plane (2θ = 34°). With the addition of up to 1.98 vol. % TiN NPs, the intensity corresponding to the basal plane of pure Mg was found to decrease. With further addition of TiN NPs (2.5 vol. %), the dominance of basal plane intensity or basal texture was still observed.

### 2.3. Microstructural Characterization

**Figure 2:**
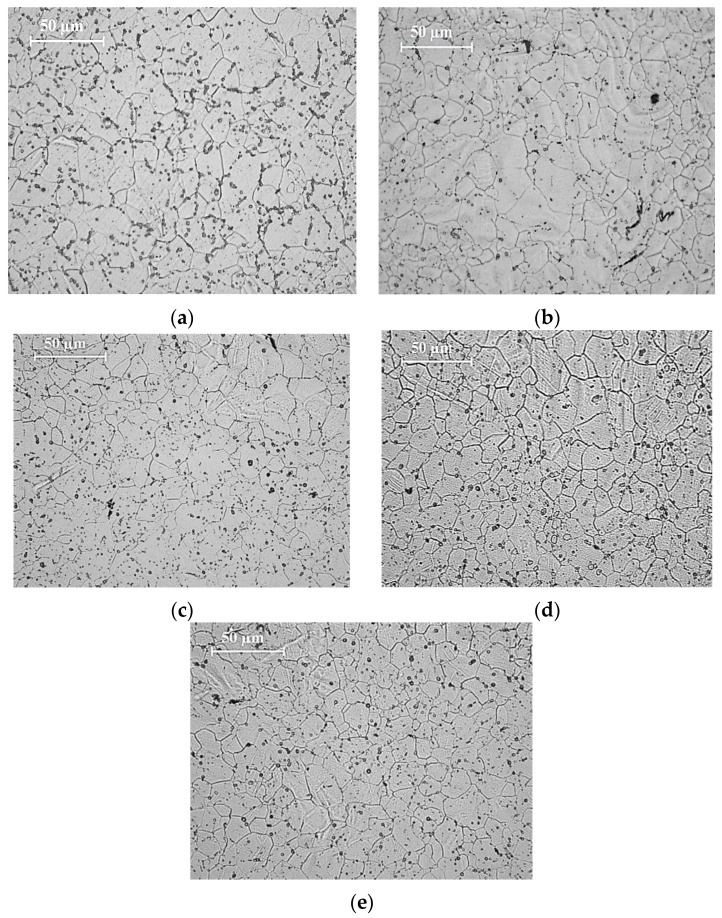
Microscopic images showing grain characteristics of: (**a**) pure magnesium; (**b**) Mg 0.58 TiN; (**c**) Mg 0.97 TiN; (**d**) Mg 1.98 TiN; and (**e**) Mg 2.5 TiN.

**Figure 3:**
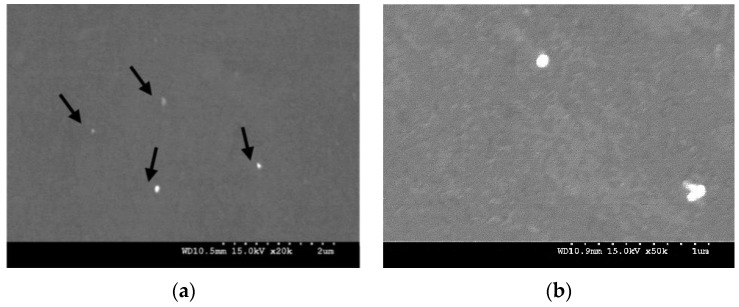
(**a**) Distribution of TiN NPs and (**b**) interfacial integrity of Mg-TiN in Mg 2.5 vol. % TiN nanocomposite.

The results of grain size measurements conducted on optical micrographs of synthesized pure Mg and Mg-TiN nanocomposites (Figure 2) are shown in Table 3. It is observed that the grain size of pure Mg decreases with the addition of TiN NPs and a minimum of ~11 μm, which is ~57% lower than that of pure Mg, was observed with Mg 2.5 vol. % TiN nanocomposite. Further, from the micrographs (Figure 3) representing the distribution of TiN NPs within the synthesized Mg-TiN nanocomposites, minimal agglomeration of TiN particulates was observed.

### 2.4. Coefficient of Thermal Expansion (CTE)

The CTE values of the synthesized pure Mg and Mg-TiN nanocomposites measured in the temperature range of 50 to 400 °C (Table 1) reveal that the average CTE values of the nanocomposites decreases with the addition of TiN NPs and thereby contribute more to the dimensional stability of pure Mg. Mg 2.5 vol. % TiN nanocomposite exhibited a minimum CTE value of ~22.61 × 10^−6^/K which is ~16% lower than that of pure Mg (27 × 10^−6^/K).

### 2.5 Microhardness Test

The hardness values of pure Mg was found to increase with the addition of TiN NPs (Table 3) and Mg 2.5 vol. % TiN exhibited a maximum hardness value of ~67 HV, which is ~26% greater than that of pure Mg and, thereby, exhibit higher constraints to localized plastic deformation.

### 2.6. Tensile Test

**Figure 4:**
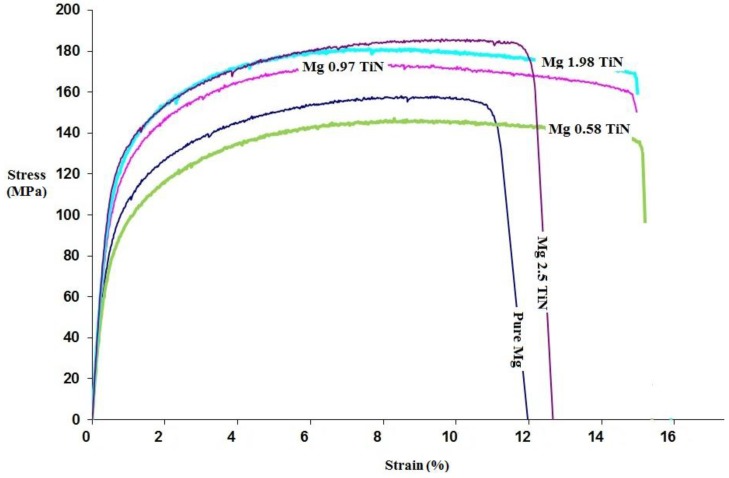
Stress-strain curve of Mg-TiN nanocomposites under tensile loading.

The room temperature tensile properties of pure Mg and Mg-TiN nanocomposites and their representative stress-strain curves are shown in Table 4 and Figure 4, respectively. The tensile strength properties of pure Mg was found to increase with the addition of ≥ (greater than or equal to) 0.97 vol. % TiN NPs and Mg 2.5 vol. % TiN exhibited the maximum 0.2% tensile yield strength (0.2% TYS) and ultimate tensile strength (UTS) of ~135 MPa and ~196 MPa, respectively, which is ~26% and ~17% greater than that of pure Mg. The tensile fracture strain of pure Mg was found to increase with up to 1.98 vol. % TiN (to ~15%) and with further addition (2.5 vol. %), the fracture strain value of pure Mg decreased to 10.6%. Further, due to simultaneous improvements in the 0.2 % TYS, UTS, and tensile fracture strain values of pure Mg observed with the addition of up to 1.98 vol. % TiN NPs, a significant increase in the energy absorbed (EA) until failure under tensile loading is noticed, indicating improvement in their damage tolerant capabilities. Among the synthesized materials, Mg 1.98 vol. % TiN nanocomposite exhibited a maximum EA value of ~26 MJ/m^3^, which is ~85% greater than that of pure Mg.

### 2.7. Compression Test

**Figure 5:**
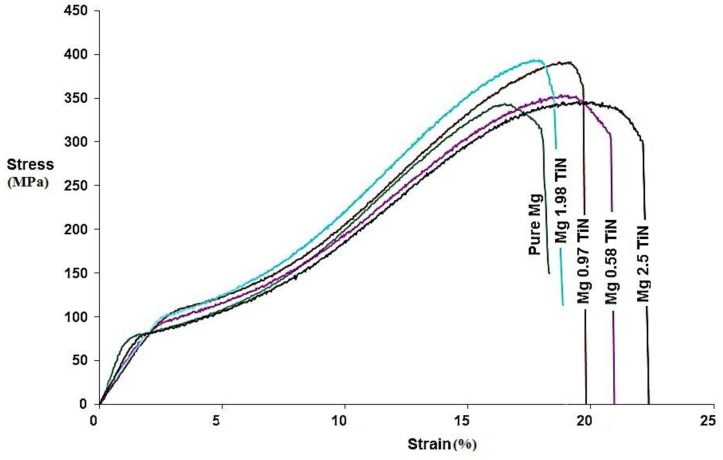
Stress-strain curve of Mg-TiN nanocomposites under compression loading.

The room temperature compression properties of pure Mg and Mg-TiN nanocomposites and their representative stress-strain curves are shown in Table 5 and Figure 5, respectively. The compression strength properties of pure Mg was found to increase with the addition of up to 1.98 vol. % TiN NPs and Mg 1.98 vol. % TiN nanocomposite exhibited the maximum 0.2% compressive yield strength (0.2% CYS) and ultimate compressive strength (UCS) of ~103 MPa and ~385 MPa, respectively, which is ~28% and ~11% greater than that of pure Mg. With further addition of TiN NPs (2.5 vol. %), the 0.2% CYS and UCS of the nanocomposite was found to decrease. Mg-TiN nanocomposites exhibited higher compressive fracture strain values (~21%) when compared to that of pure Mg (~18.5%). Further, the energy absorbed (EA) until failure under compressive loading of pure Mg was found to increase due to the presence of TiN NPs and Mg 0.97 TiN nanocomposite exhibited a maximum EA value of ~43 MJ/m^3^, which is ~15% greater than that of pure Mg.

## 3. Discussion

### 3.1. Microstructural Characteristics

**Figure 6:**
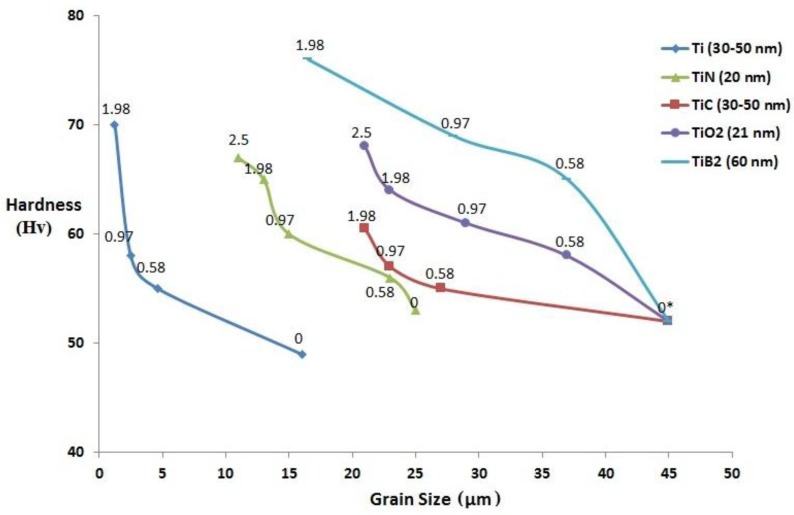
Influence of volume fraction and type of Ti nanoparticulates on the grain size and microhardness values of pure Mg. Volume fraction of reinforcements are utilized as data labels and 0 indicate hardness values of pure Mg utilized for comparison in the case of Mg-TiC, Mg-TiB_2_, and Mg-TiO_2_ nanocomposites.

During recrystallization, when compared to the presence of large sized reinforcements, ultrafine second phase reinforcements restrict the grain growth of metal matrix more significantly [14]. Figure 6 shows the influence of volume fraction and type of Ti NPs (metal and ceramics) on the grain size of pure Mg. The grain size of pure Mg was found to decrease with the addition of NPs which is due to (a) the ability of NPs to nucleate Mg grains during recrystallization and (b) restriction in the grain growth of pure Mg due to grain boundary pinning by NPs. It is observed that among the Mg nanocomposites containing Ti (metal) [15] and ceramics of Ti, TiX (where X = C [16], O_2_ [17], N (present study), B_2_ [18]) NPs, Mg-Ti nanocomposites exhibited lowest grain size of ~1.25 µm with 1.98 vol. % Ti addition and this may be due to good wetting of Ti by the molten Mg matrix [14]. In the present study, among the synthesized Mg-TiN nanocomposites, Mg 2.5 vol. % TiN exhibited a minimum grain size of ~11 µm, which is ~57% lower than that of pure Mg. The fundamental principle behind the ability of ultrafine particulates within the metal matrix to nucleate recrystallized grains and inhibit grain growth has been established already [19,20].

The effects of TiN NPs on the crystallographic orientation of pure Mg were analyzed utilizing X-ray diffraction studies. Along the transverse direction of the extruded Mg samples, dominance of prismatic intensity (2θ = 32°) was observed indicating that any of the prismatic planes is perpendicular to the extrusion direction [16,17,19]. Along the longitudinal direction, the intensity corresponding to the basal plane (2θ = 34°) of hot extruded pure Mg was found to be the maximum indicating strong basal texture with most of the basal planes parallel to the extrusion direction, which is commonly found in wrought Mg materials [16,19,21]. With the addition of up to 1.98 vol. % TiN NPs, the basal plane intensity of pure Mg was found to decrease indicating that the basal planes are no longer parallel to the extrusion direction. This similar randomization in the texture of pure Mg with the addition of certain critical quantities of NPs, such as (0.58 and 0.97) vol. % TiB_2_, (0.58 and 0.97) vol. % TiC and (0.58, 0.97, 1.98) vol. % TiO_2_ was observed previously [16,17,19]. With further addition of TiN NPs (2.5 vol. %), the basal plane intensity of pure Mg was found to increase and thereby exhibiting strong basal texture (I_basal_/I_max_ = 1) similar to that of monolithic pure Mg. Unlike ceramics of Ti, addition of Ti (metal) NPs did not contribute to the textural changes of pure Mg [15].

### 3.2. Mechanical Properties

#### 3.2.1. Microhardness

In the present study, the hardness value of pure Mg was found to increase with the addition of TiN NPs and Mg 2.5 TiN exhibited the maximum hardness value of ~67 HV. Figure 6 shows the influence of the volume fraction and type of Ti NPs (metal and ceramics) on the microhardness values of pure Mg. Within the Mg-Ti based nanocomposite systems such as Mg-Ti, Mg-TiB_2_, Mg-TiC, and Mg-TiO_2_, it is observed that the hardness value of pure Mg increases with (a) increasing volume fraction of NPs and (b) decrease in the grain size. A similar trend in the microhardness values was observed in the Mg-TiN system. Among the Mg nanocomposites, Mg 1.98 vol. % TiB_2_ exhibited the maximum hardness value of 76 HV which may be due to (a) the presence of harder TiB_2_ NPs of hardness value ~ 960 HV or ~ 34 GPa [22] (Table 6), (b) relatively larger NPs size (~60 nm), which may assist in more effective load bearing creating constraint to localized plastic deformation, and (c) for NPs of size less than 80 nm, dispersion of NPs is inversely proportional to its size and among the NPs, TiB_2_ (60 nm) has more possibility for relatively more uniform dispersion within the Mg metal matrix [23].

#### 3.2.2. Tensile Properties

In the present study, among the synthesized Mg-TiN nanaocomposites, Mg 2.5 vol. % TiN exhibited the maximum 0.2% TYS and UTS of ~135 MPa and ~196 MPa, respectively. The strength of pure Mg was found to increase with the addition of ≥ (greater than or equal to) 0.97 vol. % TiN NPs. The major mechanisms contributing to the strengthening of particulate reinforced metal matrix nanocomposites (MMNCs) are [29] (a) Orowan strengthening from dislocation bowing of NPs, (b) Hall-Petch strengthening from grain refinement, (c) Forest strengthening resulting from the mismatch in the coefficient of thermal expansion values between the metal matrix and NPs, (d) Taylor strengthening due to the mismatch in the modulus values between the metal matrix and NPsm, and (e) strengthening due to load bearing of the NPs.

The presence of NPs within the metal matrix tends to form dislocation loops around the NPs and thereby offer resistance to dislocation movement and assist in strengthening of MMNCs. This strengthening mechanism observed in MMNCs is termed as Orowan strengthening. The strength improvement due to the Orowan effect of particulate reinforcement within the Mg matrix is given by the Orowan–Ashby equation as shown in Equation (1) [30]:

(1) $$\sigma_{Orowan}=\frac{0.13\text{Gb}}{\lambda }\ln\frac{r\;}{b}$$

where, G is the shear modulus of Mg (17.3 GPa) [31]; b is the Burgers vector of Mg (3.21 × 10^−10^ m) [32]; *r* and *d_p_* are the average radius and diameter of NPs, respectively. The interparticulate spacing (λ) between the NPs within the Mg metal matrix is given by Equation (2) [33,34]:

(2) $$\lambda =d_{p\;}\left[{\left(\frac{1}{2\;V_{p}\;}\right)}^{\frac{1}{3}}-1\right]$$

where, *V_p_* is the volume fraction addition of Ti-based NPs.

The influence of particulate size and volume fraction of reinforcements on the interparticulate spacing (λ) and Orowan strengthening contribution (σ_Orowan_) towards 0.2%TYS of Mg-Ti based nanocomposites is shown in Table 7. In the case of Mg 0.58 TiN nanocomposite, to realize σ_Orowan_ of ~37 MPa, TiN NPs need to be uniformly distributed with an average interparticulate spacing of ~68 nm. With a further increase in the volume fraction of TiN NPs, the value of λ decreases and, for Mg 2.5 vol. % TiN, a λ of 34.20 nm is to be achieved to realize an σ_Orowan_ of ~73 MPa. Experimentally, it is difficult to achieve uniform distribution of NPs with λ closer to the theoretically-predicted values in the range of nm scale lengths. The effectiveness of synthesis methodology in the dispersion of NPs within the metal matrix play a vital role in composite technology and, failing which clustering of NPs take place, that (a) affecting mean effective particulate size within the metal matrix leading to distribution of particle sizes and (b) randomized λ values within the metal matrix may be observed. In the case of adopted synthesis methodology (disintegrated melt deposition technique, DMD), stirring parameters play a vital role in controlling the clustering effects within the composites. The density of Ti-based reinforcements utilized for synthesizing Mg nanocomposites (Table 6) was found to be in the range of ~4.5 to 5.25 g/cc indicating minimal or no significant changes and with the adopted synthesis methodology utilizing constant stirring parameters (465 rpm for 6 min) make the study a fair comparison among the Mg-Ti based nanocomposites. Theoretically, for a constant volume fraction, σ_Orowan_, in the case of Mg-TiN and Mg-TiO_2_ materials, was found to be the maximum due to the presence of lower particulate size of ~20 nm and ~21 nm, respectively. Previously, the authors studied the effect of synthesis methodology (DMD) on the dispersion of TiO_2_ NPs by experimentally measuring the value of λ. It was found that the presence of TiO_2_ NPs of size ~21 nm contributed only ~4 MPa and ~7 MPa to the 0.2% TYS in the case of Mg (1.98 and 2.5) vol. % TiO_2_ nanocomposites, respectively. Therefore, in the case of Mg-TiN nanocomposites with TiN NPs of size and density (~20 nm and 5.22 g/cc) closer to that of TiO_2_ NPs (~21 nm and 4.23 g/cc), the Orowan strengthening contribution (σ_Orowan_) may be considered almost negligible. 

Grain size of a material is inversely proportional to its tensile yield strength (TYS) and the strengthening effect relating the grain size to the strength of the material is termed as Hall-Petch Strengthening. The following equation describes the Hall-Petch equation:

(3) $$\sigma_{Hall-Petch}=KD^{-0.5}$$

where, *K* is the Hall-Petch coefficient of Mg (280 MPa μm^1/2^) and *D* is the average grain size of synthesized Mg nanocomposites. Further, the relationship between reinforcement particulate size, volume fraction of particulates, and grain size of composites is given by Zener equation, Equation (4) [35]:

(4) $$d_{m}=\;\frac{4\;\alpha \;d_{p}}{3\;v_{p}}$$

where, α is proportionality constant; $d_{p}$ is the average diameter of the NPs; $v_{p}$is the volume fraction of NPs; and $d_{m}$ is the grain size of the metal matrix.

Table 8 shows the contribution of Hall-Petch (σ_Hall-Petch_) and Orowan strengthening (σ_Orowan_) due to the presence of Ti-based NPs to the TYS of pure Mg. Among the Mg-Ti based nanocomposites, σ_Hall-Petch_ values corresponding to Ti (metal) NPs were found to be the maximum with Mg 1.98 vol. % Ti exhibiting σ_Hall-Petch_ as high as ~250.4 MPa. This increase in the σ_Hall-Petch_ contribution observed with Mg-Ti materials is attributed to the significant grain refinement with Mg 1.98 vol. % Ti exhibiting the lowest grain size of ~1.25 μm. The σ_Hall-Petch_ was found to increase with (a) lower particulate size and (b) higher volume fraction addition of NPs. Among the ceramics of Ti, TiN was more effective towards σ_Hall-Petch_ contribution and Mg 2.5 vol. % TiN exhibited the maximum σ_Hall-Petch_ contribution of ~84 MPa.

Strengthening due to load bearing ($\sigma_{LT}$) or transferring of applied load to NPs within the metal matrix is given by the following equation:

(5) $$\sigma_{LT}=0.5\;v_{p\;}\sigma_{Mg}$$

where, $\sigma_{Mg}$ is the experimental 0.2% TYS of pure Mg; and $\sigma_{LT}$ depends on the volume fraction of NPs added to the metal matrix. In the case of Mg (0.58, 0.97, 1.98, and 2.5) vol. % TiN nanocomposites, $\sigma_{LT}$ was found to be ~0.23 MPa, ~0.38 MPa, ~0.79 MPa, and ~1MPa, respectively, which are insignificant and, therefore, the contribution of strengthening due to load bearing is negligible in the case of low-volume fraction reinforced Mg MMNCs.

Forest strengthening, or strengthening due to CTE mismatch ($\sigma_{CTE}$) between the Mg matrix and NPs, leads to increase in the dislocation density by generation of dislocations nearby the NPs. Taylor strengthening, or strengthening due to mismatch in the modulus values between the Mg matrix and NPs $(\sigma_{EM}),\;$leads to formation of geometrically-necessary dislocations (GND) due to straining or the presence of external load, and is profound only with higher volume fraction NPs addition. The CTE and modulus values of Ti-based reinforcements are shown in Table 6. Previously, it was experimentally found that in the case of MMNCs with low volume fraction (< 10 vol. %) NPs of size less than 80 nm, the Forest strengthening contribution is almost negligible [23,36,37]. When compared to the contributions of other strengthening mechanisms, the contributions from Taylor strengthening is also considered negligible [29,38].

**Figure 7:**
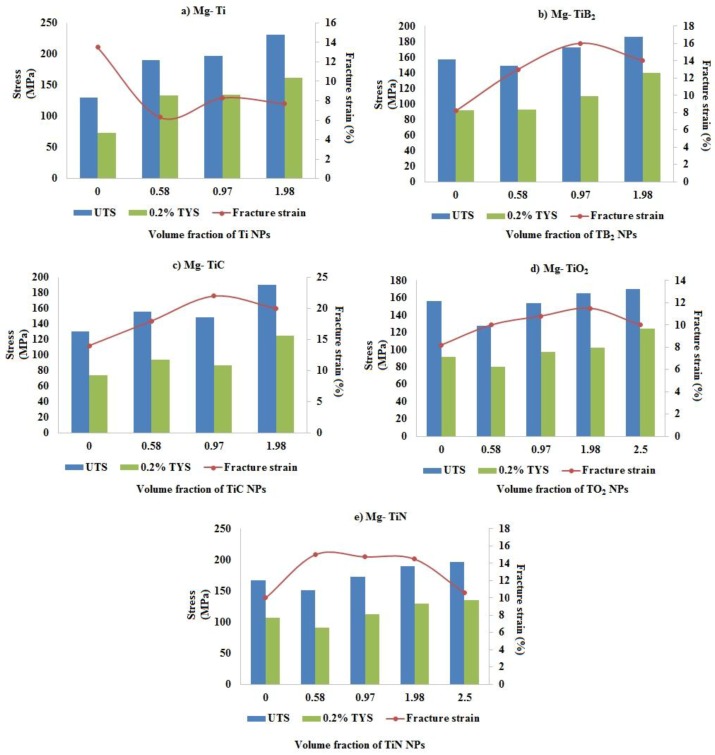
Influence of volume fraction and type of Ti nano-reinforcement on the tensile properties of pure Mg. (**a**) Mg-Ti; (**b**) Mg-TiB_2_; (**c**) Mg-TiC; (**d**) Mg-TiO_2_; (**e**) Mg-TiN.

Figure 7 shows the influence of volume fraction and type of Ti reinforcement on the strength and fracture strain values of Mg nanocomposites under tensile loading. It is observed that presence of Ti (ceramic) NPs within the Mg metal matrix significantly improve the tensile fracture strain value of pure Mg when compared to that of Ti (metal) NPs and among the Mg MMNCs containing Ti-based reinforcements, Mg 0.97 TiC exhibited the maximum tensile fracture strain value of ~22%. Improvement in the tensile fracture strain values in the case of Mg MMNCs containing Ti ceramics (brittle phases) NPs is due to the ability of the ultrafine particulates to weaken the basal texture of pure Mg which is observed through X-ray diffraction studies. In the present study, Mg (0.58, 0.97, and 1.98) TiN exhibited the maximum tensile fracture strain values of ~15%, which is ~50% greater than that of pure Mg (~10%). With further addition of TiN NPs (2.5 vol. %), strong basal texture similar to that of pure Mg was observed with a decrease in its tensile fracture strain value to ~10.5%.

**Figure 8:**
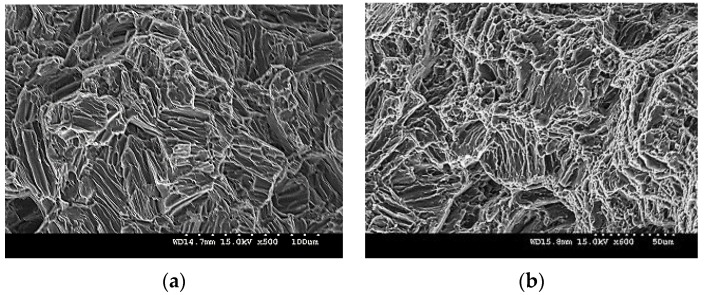
Fractographs of (**a**) pure Mg and (**b**) Mg 2.5 TiN under tensile loading.

The fracture surfaces of Mg-TiN nanocomposites are discussed with representative fractograph images of pure Mg and Mg 2.5 TiN nanocomposite after tensile loading as shown in Figure 8. For all pure Mg and Mg-TiN nanocomposites, a typical cleavage mode of fracture is observed which indicates that the fracture behavior of Mg-TiN materials is greatly controlled by pure Mg matrix.

#### 3.2.3. Compressive Properties

**Figure 9:**
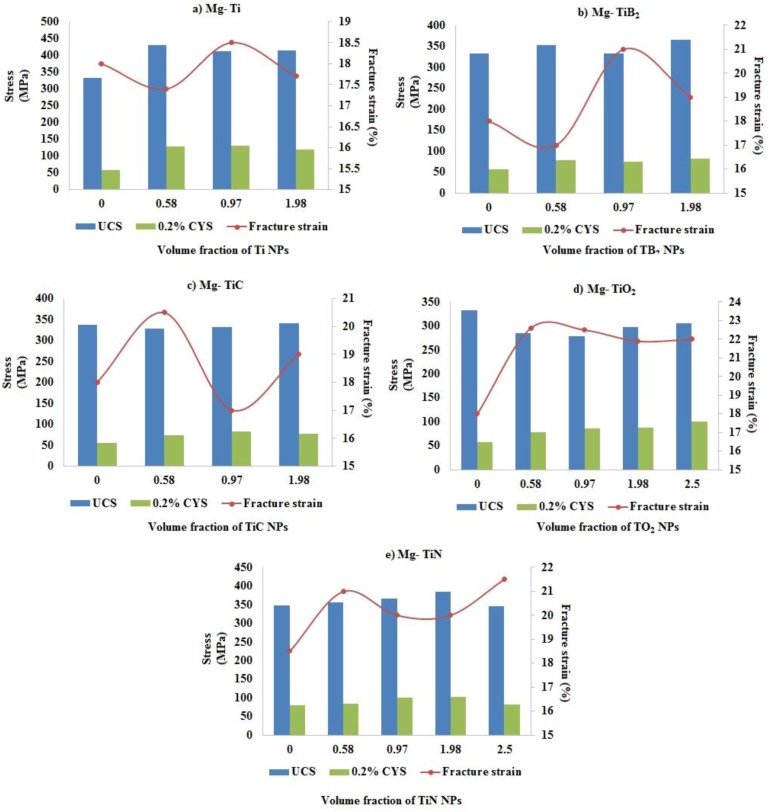
Influence of volume fraction and type of Ti nano-reinforcement on the compressive properties of pure Mg. (**a**) Mg-Ti; (**b**) Mg-TiB_2_; (**c**) Mg-TiC; (**d**) Mg-TiO_2_; (**e**) Mg-TiN.

Figure 9 shows the influence of Ti-based NPs on the compressive properties of pure Mg. The error bars with lower and upper limits indicate the 0.2% CYS and UCS of Mg-Ti-based nanocomposites, respectively. The presence of Ti (metal) NPs within the Mg metal matrix was found to significantly improve the strength properties of pure Mg under compression loading. The improvement in the strength properties of Mg-Ti (metal) nanocomposites may be due to significant grain refinement contributing to Hall-Petch strengthening. Mg 0.97 vol. % Ti exhibited the highest compression strength properties of ~130 MPa (0.2% CYS) and ~413 MPa (UCS), respectively. The compressive strain values of Mg-Ti (metal) nanocomposites were found to be lower than that of pure Mg (~18%). When compared to Ti (metal) NPs, the presence of Ti (ceramic) NPs significantly improved the compressive fracture strain values of pure Mg. Among the Ti (ceramics) NPs, Mg 1.98 vol. % TiN exhibited the maximum compressive strength properties of ~103 MPa (0.2%CYS) and ~385 MPa (UCS), respectively, and Mg (0.58 and 0.97) TiC exhibited the maximum compressive fracture strain value of ~22.5%.

In the present study, the strength and fracture strain values of Mg-TiN nanocomposites under compression loading was found to increase with the addition of up to 1.98 vol. % TiN and Mg 1.98 vol. % TiN exhibited the maximum 0.2% CYS, UCS, and compressive fracture strain values of ~103 MPa, ~385 MPa, and ~20%, respectively. The increase in the strength properties of Mg-TiN nanocomposites under compression is due to the contribution of Hall-Petch strengthening and Mg 1.98 vol. % TiN with a lower grain size of ~13 μm exhibited the highest compression strength properties. With further addition of TiN NPs (2.5 vol. %), compressive fracture strain values of the nanocomposite was found to increase with decrease in the strength properties (0.2% CYS and UCS) which may be due to: (a) presence of possible agglomeration sites within the nanocomposite and (b) strong basal texture observed with Mg 2.5 vol. % TiN similar to that of pure Mg. From Table 7, it can be observed that it is relatively difficult to uniformly disperse 2.5 vol. % TiN NPs of size ~20 nm within the Mg matrix with an interparticulate spacing (λ) of ~34.20 nm when compared to that of lower volume fraction of Mg (0.58, 0.97, 1.98) vol. % TiN nanocomposites.

**Figure 10:**
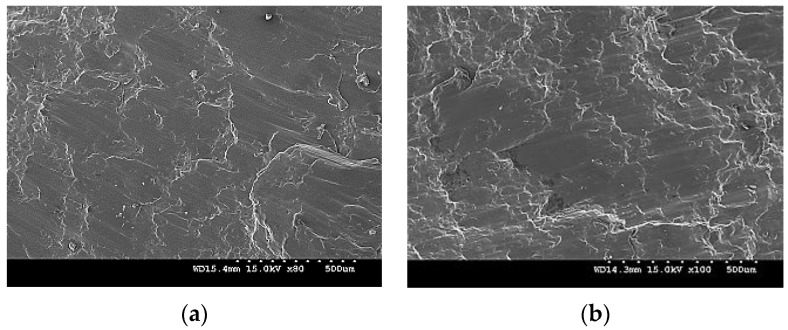
Fractographs of (**a**) Pure Mg and (**b**) Mg 2.5 TiN under compressive loading.

The fracture surfaces of Mg-TiN nanocomposites are discussed with representative fractograph images of pure Mg and Mg 2.5 TiN nanocomposite after compressive loading, as shown in Figure 10. It is observed that failure in pure Mg and Mg-TiN nanocomposites occurred at 45° with respect to the compression loading axis and their representative fractographs indicate the presence of shear bands. 

The tensile deformation of Mg materials is governed by slip mode with basal slip as the most dominant mechanism [18]. However, under compression, the initial deformation of Mg materials is by tensile twinning as the critically-resolved shear stress required to initiate basal slip under compression is more than that of twinning [18]. The directional nature of twinning makes Mg materials exhibit large anisotropy when deformed under different stress states and initial textures [39,40]. A way to capture the anisotropy in Mg materials is by measuring tensile-compression asymmetry (TCA) value which is given by σ_y,t_/σ_y,c_ where σ_y,t_ and σ_y,c_ are the uniaxial 0.2% yield strength of Mg materials under tensile and compression loading, respectively. In the case of Mg-Ti (metal) nanocomposites, with addition of 1.98 vol. % Ti, TCA value increased significantly, which is representative from its tensile fracture strain value of only ~7.7%. Strengthening in the case of Mg-Ti (metal) nanocomposites was observed with significant decrease in the tensile fracture strain values. Whereas in the case of Mg-Ti (ceramic) NPs, the TCA value was found to decrease with up to certain critical quantity of NPs addition and with further addition of NPs, the TCA value of Mg nanocomposite was found to, once again, increase with the reduction in the tensile fracture strain values. Table 9 shows the TCA values of Mg-TiN nanocomposites. It is observed that with addition of up to 1.98 vol. % TiN, TCA value of Mg-TiN nanocomposites was found to decrease and Mg 0.58 TiN exhibited a minimum TCA value of ~1.09. Weakening of strong basal texture of pure Mg which allows non-basal cross-slip to occur is possible in the case of Mg (0.58, 0.97, and 1.98) vol. % TiN nanocomposites and under tensile loading, only marginal improvement in the 0.2% TYS is possible. For this reason, the 0.2% TYS of Mg 0.58 TiN was found to be ~91 MPa, which is ~15% lower than that of pure Mg exhibiting a higher tensile fracture strain value of ~15%. Among the Mg-TiN nanocomposites, Mg (0.58, 0.97, and 1.98) vol. % TiN exhibited the maximum tensile fracture strain values of ~14 to 15%. With further addition of TiN NPs (2.5 vol. %), the TCA value of pure Mg was found to increase to 1.64 which is representative from its tensile fracture strain value of only ~10.6% similar to that of pure Mg (~10%).

## 4. Materials and Methods

### 4.1. Materials

In the present study, > 99.9% pure elemental magnesium turnings supplied by ACROS organics, New Jersey, USA, were used as the base material and the required amount of pure titanium nitride (TiN) powder of size ~20 nm and purity > 99.2% supplied by US Research Nanomaterials, Inc. (Houston, TX, USA), was used as the reinforcement phase.

### 4.2. Processing

#### 4.2.1. Primary Processing

Pure magnesium and Mg (0.58, 0.97, 1.98, and 2.5) vol. % TiN nanocomposites were synthesized by a disintegrated melt deposition technique. Within a graphite crucible, pure magnesium turnings and TiN nanoparticulates (NPs) were placed in a multilayered sandwich fashion and superheated to 750 °C under an argon gas atmosphere using a resistance heating furnace. For uniform distribution of NPs within the metal matrix, the superheated slurry was stirred at 465 rpm for 6 min using a twin-blade stirrer with a pitch of 45°. ZIRTEX 25 coating was applied on the stirrer to avoid iron contamination of the molten metal. After stirring, the molten metal was then poured into the mold, under the influence of gravity, through a 10 mm hole in the crucible. Before entering the mold, the molten metal was disintegrated by two jets of argon gas oriented normal to the melt stream. The flow rate of argon was maintained at 25 lpm and an ingot of 40 mm diameter was obtained. For synthesizing pure magnesium, no NPs were added and the above steps were followed. The 40 mm diameter ingots obtained were then machined to a diameter of 36 mm for hot extrusion.

#### 4.2.2. Secondary Processing

Before extrusion, the machined ingots were soaked at 400 °C for 1 h in a constant temperature furnace. Using a 150 T hydraulic press, hot extrusion was carried out at 350 °C die temperature, with an extrusion ratio of 20.25:1 for obtaining rods of 8 mm in diameter. The samples from the extruded rods were used for characterization, as detailed in the next section.

### 4.3. Materials Characterization

#### 4.3.1. Density Measurements

Density of extruded pure Mg and Mg-TiN nanocomposites in polished condition was measured using Archimedes principle. Three samples from different ends of the extruded rods were accurately weighed in air and then immersed in distilled water. An A and D ER-182A electronic balance with an accuracy of 0.0001 g was used for measuring the weights. Using the rule of mixtures principle, the theoretical densities of the synthesized Mg materials were calculated. Porosity values of the synthesized Mg materials were calculated utilizing Equation (6):

(6) $$Porosity=\;\frac{\rho_{th}-\;\rho_{exp}}{\rho_{th}-\;\rho_{air}}\;\times 100$$

where $\rho_{th}$ is the theoretical density (g/cm^3^); $\rho_{exp}$ is the experimental density in (g/cm^3^); and $\rho_{air}$ is the density of air (0.001225 g/cm^3^).

#### 4.3.2. X-ray Diffraction Studies

The extruded pure magnesium and Mg-TiN nanocomposite samples were exposed to Cu Kα radiation of wavelength λ = 1.54056 Å with a scan speed of 2 °/min by using an automated Shimadzu lab-X XRD-6000 diffractometer (Shimadzu, Kyoto, Japan). The Bragg angles and the values of the interplanar spacing, *d*, obtained were subsequently matched with the standard values of Mg, TiN and related phases. Further, the basal plane orientation of Mg-TiN nanocomposites was analyzed based on the XRD peaks obtained from experiments carried out in the directions both parallel and perpendicular to the extrusion axis.

#### 4.3.3. Microstructural Characterization

To investigate on TiN reinforcement distribution and grain size of pure magnesium and Mg-TiN nanocomposites, the microstructural characterization studies were conducted on metallographically-polished extruded samples and a Hitachi S-4300 field emission scanning electron microscope (FESEM) (Hitachi, Tokyo, Japan), an Olympus metallographic optical microscope (Tokyo, Japan) and Scion image analysis software (Scion, Sacramento, CA, USA) were utilized. For every nanocomposite sample, five micrographs were utilized for more accurate estimation of grain size.

#### 4.3.4. Coefficient of Thermal Expansion (CTE)

By using a thermo-mechanical analysis instrument “LINSEIS TMA PT 1000LT” (Linseis, Princeton Junction, NJ, USA), the coefficient of thermal expansion values of pure magnesium and Mg-TiN nanocomposites were determined. Heating rate of 5 °C/min was maintained with an argon flow rate of 0.1 lpm. By using an alumina probe, the displacement of the test samples (each of 5 mm length) was measured as a function of temperature (323 to 623 K).

#### 4.3.5. Microhardness Test

Using a Shimadzu HMV automatic digital microhardness tester (Shimadzu, Kyoto, Japan) with a Vickers indenter (square based pyramidal shaped diamond indenter with a phase angle of 136°), the microhardness tests were conducted on flat and metallographically-polished specimens. An indenting load of 25 gf for a dwell time of 15 s was used. The testing was performed as per ASTM E384-11e1.

#### 4.3.6. Tensile Test

In accordance with ASTM E8/E8M-15a, the smooth bar tensile properties of pure magnesium and Mg-TiN nanocomposites were determined at ambient temperature. The tensile tests were conducted on round tension test specimens of 5 mm diameter and gauge length 25 mm using a fully automated servo-hydraulic mechanical testing machine, MTS-810. The strain rate was set to 1.693 × 10^−4^ s^−1^ and an Instron 2630-100 series extensometer (Instron, Singapore) was used to measure the fracture strain. For each composition, five samples were tested to ensure repeatable values.

#### 4.3.7. Compression Test

In accordance with ASTM E9-09, the smooth bar compressive properties of the extruded pure magnesium and Mg-TiN samples were determined at ambient temperature, using a MTS-810 testing machine (Instron, Singapore) with a strain rate of 8.334 × 10^−5^ s^−1^. The test specimens of 8 mm diameter and length to diameter ratio l/*d* ~ 1 were used. For each composition, five samples were tested to ensure repeatable values.

#### 4.3.8. Fracture Behavior

To provide an insight into the various possible fracture mechanisms operating during the tensile and compression loading of the samples, characterization of fractured surfaces of tensile and compression samples were successfully investigated using Hitachi S-4300 FESEM (Hitachi, Tokyo, Japan).

## 5. Conclusions

In the present study, Mg (0.58, 0.97, 1.98, and 2.5) vol. % TiN nanocomposites are synthesized by a disintegrated melt deposition technique followed by hot extrusion. An attempt is made to study and compare the effect of type of Ti based (metal and ceramic) NPs, their size, and volume fraction addition on the microstructural and mechanical properties of pure Mg.

The following are the primary conclusions of the present study:

(a) Utilizing the adopted synthesis methodology (disintegrated melt deposition technique followed by hot extrusion), near dense Mg-Ti based nanocomposites containing low-volume fraction Ti (metal) and ceramics of Ti NPs can be synthesized. It is observed that with a marginal increase in the density of pure Mg, the presence of Ti-based NPs significantly improves its mechanical properties.
(b) Microstructural characterization indicate significant grain refinement of pure Mg with the addition of TiN NPs and Mg 2.5 vol. % TiN exhibited a minimum grain size of ~11 μm, which is 57% lower than that of pure Mg. Among the Mg-Ti based nanocomposites, Mg 1.98 vol. % Ti (metal) nanocomposites exhibited the lowest grain size of ~1.5 μm.
(c) Microhardness values of pure Mg increases with the addition of TiN NPs and Mg 2.5 vol. % TiN exhibited the maximum hardness value of ~67 HV, which is 26% greater than that of pure Mg. Among the Mg-Ti based nanocomposites, Mg 1.98 vol. % TiB_2_ exhibited the maximum hardness value of ~76 HV.
(d) Room temperature tensile properties of Mg-TiN nanocomposites indicate an increase in the strength properties of pure Mg with addition of ≥ (greater than or equal to) 0.97 TiN NPs. Mg 2.5 vol. % TiN nanocomposites exhibited the maximum 0.2%TYS and UTS of ~135 MPa and ~196 MPa, respectively, which are ~26% and ~17% greater than that of pure Mg.
(e) X-ray diffraction studies indicated that addition of up to 1.98 vol. % TiN NPs has the ability to modify the basal texture of hot extruded pure Mg. The tensile fracture strain values of pure Mg was found to increase with up to 1.98 vol. % TiN NPs addition and Mg (0.58, 0.97, 1.98) vol. % TiN exhibited the maximum tensile fracture strain values of ~15%. With further addition of TiN NPs (2.5 vol. %), strong basal texture of pure Mg was observed and the tensile fracture strain values of Mg 2.5 vol. % TiN was found to decrease to ~10.5%. Further, critical quantity of Ti (ceramic) NPs contribute to modifying the basal texture of pure Mg and thereby enhance the fracture strain values, whereas no textural changes of pure Mg reinforced with metallic Ti NPs was observed.
(f) Among the major strengthening mechanisms of Mg MMNCs containing Ti-based NPs, Hall-Petch strengthening contribution was found to play a vital role. Mg-Ti (metal) nanocomposites exhibited the maximum 0.2%TYS with significant Hall-Petch contributions and Mg 1.98 vol. % Ti possessed 0.2% TYS as high as ~162 MPa. Among the Mg-Ti (ceramic) nanocomposites, Mg 1.98 TiB_2_ exhibited 0.2%TYS as high as ~140 MPa.
(g) Room temperature compression properties of Mg-TiN nanocomposites indicate that, with the addition of up to 1.98 vol. % TiN the 0.2% CYS, UCS and compressive fracture strain values of pure Mg was found to increase. Mg 1.98 vol. % TiN exhibited the highest 0.2CYS, UCS and compressive fracture strain values of ~103 MPa, ~385 MPa, and ~21%, respectively, which are ~28%, ~11%, and ~7% greater than that of pure Mg. With further addition of TiN (2.5 vol. %) NPs, the compression strength properties were found to decrease with 0.2%CYS and UCS of ~82 MPa and ~342 MPa, respectively. Mg-Ti (ceramic) nanocomposites were found to exhibit higher compressive fracture strain, whereas strengthening was predominantly observed in Mg-Ti (metal) nanocomposites.
(h) Further, addition of up to 1.98 vol. % TiN NPs aids in minimizing the TCA value of pure Mg and Mg (0.58 and 0.97) TiN exhibited the minimum TCA value of ~1.10.

## Figures and Tables

**Table 1:** Density and coefficient of thermal expansion (CTE) measurements of pure magnesium and synthesized Mg-TiN nanocomposites.

Sl. No	Material	Reinforcement		Density Measurements			CTE (×10^−6^/K)
		(Wt %)	(Vol %)	Theoretical Density (g/cm^3^)	Experimental Density (g/cm^3^)	Porosity (%)	
1	Mg	Nil	Nil	1.7400	1.7356	0.2530	27.00
2	Mg 0.58 TiN	1.78	0.58	1.7612	1.7589	0.1322	25.54
3	Mg 0.97 TiN	2.95	0.97	1.7750	1.7695	0.3382	24.63
4	Mg 1.98 TiN	5.9	1.98	1.8125	1.8050	0.4123	24.58
5	Mg 2.5 TiN	7.37	2.5	1.8315	1.8225	0.4917	22.61

**Table 2:** X-ray diffractogram results of as-extruded Mg-TiN nanocomposites.

Material	Section	Plane	I/I_max_
Pure Mg	T	10–10 Prism	1.000
		0002 Basal	0.104
		10–11 Pyramidal	0.278
	L	10–10 Prism	0.136
		0002 Basal	1.000
		10–11 Pyramidal	0.764
Mg 0.58 TiN	T	10–10 Prism	1.000
		0002 Basal	0.131
		10–11 Pyramidal	0.295
	L	10–10 Prism	0.166
		0002 Basal	0.551
		10–11 Pyramidal	1.000
Mg 0.97 TiN	T	10–10 Prism	1.000
		0002 Basal	0.674
		10–11 Pyramidal	0.685
	L	10-10 Prism	0.195
		0002 Basal	0.813
		10–11 Pyramidal	1.000
Mg 1.98 TiN	T	10–10 Prism	1.000
		0002 Basal	0.596
		10–11 Pyramidal	0.661
	L	10–10 Prism	0.147
		0002 Basal	0.842
		10-11 Pyramidal	1.000
Mg 2.5 TiN	T	10–10 Prism	1.000
		0002 Basal	0.460
		10–11 Pyramidal	0.464
	L	10–10 Prism	0.130
		0002 Basal	1.000
		10–11 Pyramidal	0.651

Notes: T and L represents XRD taken along transverse and longitudinal sections of Mg-TiN samples; I is the XRD intensity from prismatic, basal, and pyramidal plane of pure Mg; I_max_ is the maximum XRD intensity from either prism, basal, and pyramidal plane.

**Table 3:** Results of microstructure and microhardness studies.

Sl. No	Material	Grain Size (× 10^−6^ m)	Aspect Ratio (× 10^−6^ m)	Microhardness (HV)
1	Mg	25.5 ± 2	1.25 ± 0.2	53 ± 1
2	Mg 0.58 TiN	23 ± 2.5 (↓9%)	1.81 ± 0.1	56 ± 1.5 (↑6%)
3	Mg 0.97 TiN	15 ± 2.5 (↓41%)	1.28 ± 0.2	60 ± 1.5 (↑13%)
4	Mg 1.98 TiN	13 ± 3.5 (↓49%)	1.32 ± 0.4	65 ± 2.5 (↑23%)
5	Mg 2.5 TiN	11 ± 3.5 (↓57%)	1.35 ± 0.4	67 ± 3 (↑26%)

**Table 4:** Results of room temperature tensile testing.

Material	0.2%TYS (Mpa)	UTS (Mpa)	Fracture Strain (%)	Energy absorbed (MJ/m^3^)
Pure Mg	107 ± 5	167 ± 7	10 ± 1	14 ± 2
Mg 0.58 TiN	91 ± 5 (↓15%)	151 ± 4 (↓10%)	15 ± 1 (↑50%)	20 ± 1 (↑43%)
Mg 0.97 TiN	112 ± 2 (↑5%)	173 ± 1 (↑4%)	15 ± 2 (↑50%)	24 ± 2.5 (↑71%)
Mg 1.98 TiN	130 ± 7 (↑21%)	190 ± 11 (↑14%)	14.5 ± 1 (↑45%)	26 ± 4 (↑85%)
Mg 2.5 TiN	135 ± 8 (↑26%)	196 ± 14 (↑17%)	10.6 ± 1.2 (↑5%)	19.8 ± 1 (↑43%)

**Table 5:** Results of room temperature compression testing.

Material	0.2% CYS (Mpa)	UCS (Mpa)	Fracture Strain (%)	Energy Absorbed (MJ/m^3^)
Pure Mg	80.4 ± 2.5	347 ± 4	18.5 ± 1.5	37.3 ± 2
Mg 0.58 TiN	83.4 ± 2 (↑4%)	355 ± 8 (↑2%)	21 (↑13%)	42.5 ± 2 (↑14%)
Mg 0.97 TiN	101 ± 3 (↑26%)	365.5 (↑5%)	20 ± 1 (↑7%)	43 ± 1 (↑15%)
Mg 1.98 TiN	103 ± 5 (↑28%)	385 ± 13 (↑11%)	20 ± 1 (↑7%)	42 ± 3 (↑13%)
Mg 2.5 TiN	82 ± 3	345 ± 1	21 ± 1.5 (↑13%)	42 ± 1 (↑12%)

**Table 6:** Properties of Ti (metal) and ceramics of Ti (TiB_2_, TiC, TiN, TiO_2_).

Reinforcement or Nanoparticulates	Properties						
	Crystal Type	Density (g/cc)	Average Particle Size Utilized in Mg MMNC (nm)	Melting point (°C)	Young’s modulus (GPa)	Vicker’s hardness (GPa)	CTE (10^−6^/K)
Ti [24]	hcp	4.5	40	1667	120	0.830–3.420	8.9
TiB_2_ [25,26]	Hexagonal	4.52	60	2790	530	34	7.9
TiC [27]	Cubic	4.93	40	3067	300–480	29–32	7.4
TiO_2_ [28]	tetragonal	4.23	21	1843	230	7–11	9
TiN [28]	Cubic	5.22	20	2930	390	24	9.35

**Table 7:** Orowan strengthening contribution towards 0.2% TYS of Ti based NPs reinforced Mg nanocomposites.

Volume Fraction (%)	Orowan Stress σ_Orowan_ (MPa) and Interparticulate Spacing λ (nm)	Reinforcement Size (in nm)			
		TiN (20 nm) (Present study)	TiO_2_ (21 nm)	Ti and TiC (40 nm)	TiB_2_ (60 nm)
0.58	σ_Orowan_	36.83	35.08	18.41	12.27
	λ	68.30	71.70	337.00	205.00
0.97	σ_Orowan_	46.25	44.05	23.12	15.41
	λ	54.40	57.10	108.00	163.00
1.98	σ_Orowan_	65.09	62.00	32.54	21.69
	λ	38.70	40.60	77.30	116.00
2.5	σ_Orowan_	73.42	69.93	NA	
	λ	34.20	36.03		

**Table 8:** Theoretical contribution of Orowan and Hall-Petch strengthening mechanisms to the 0.2%TYS of Mg-Ti-based nanocomposites.

Material	Volume Fraction (%)	Experimental 0.2%TYS (MPa)	σ_Orown_ (MPa)	σ_Hall-Peth_ (MPa)
Mg-Ti	0.58	134.00	18.41	130.00
	0.97	135.00	23.12	177.10
	1.98	162.00	32.54	250.40
Mg-TiB_2_	0.58	93.00	12.27	46.03
	0.97	110.00	15.41	52.91
	1.98	140.00	21.69	68.93
Mg-TiC	0.58	94.00	18.41	53.88
	0.97	87.00	23.12	58.34
	1.98	125.00	32.54	61.10
Mg-TiO_2_	0.58	80.00	35.08	46.03
	0.97	97.00	44.05	52.00
	1.98	102.00	62.00	58.38
	2.50	124.00	69.93	61.10
Mg-TiN (Present study)	0.58	91.00	36.83	58.38
	0.97	112.00	46.25	72.30
	1.98	130.00	65.09	77.65
	2.50	135.00	73.42	84.42

**Table 9:** TCA value of synthesized Mg-TiN nanocomposites.

Material	0.2% TYS	0.2% CYS	TCA
Pure Mg	107 ± 5	80.4 ± 2.5	1.33
Mg 0.58 TiN	91 ± 5	83.4 ± 2	1.09
Mg 0.97 TiN	112 ± 2	101 ± 3	1.10
Mg 1.98 TiN	130 ± 7	103 ± 5	1.26
Mg 2.5 TiN	135 ± 8	82 ± 3	1.64

## Abbreviations

The following abbreviations are used in this manuscript:

$\sigma_{Mg}$: Tensile yield strength of monolithic pure Mg (MPa)
$\sigma_{Orowan}$: Orowan strengthening contribution (MPa)
$\sigma_{Hall-Petch}$: Hall-Petch strengthenig contribution (MPa)
$\sigma_{CTE}$: Forest strengthening contribution (MPa)
$\sigma_{EM}$: Taylor strengthening contribution (MPa)
$\sigma_{LT}$: Strengthening due to load transfer (MPa)
$\rho_{th}$: Theoretical density (g/cc)
$\rho_{exp}$: Experimental density (g/cc)
0.2% TYS: 0.2% Tensile Yield Strength (MPa)
0.2% CYS: 0.2% Compressive Yield Strength (MPa)
CTE: Coefficient of thermal expansion (μ/K)
$d_{p\;}$: Average Diameter of the NPs (m)
MMNCs: Metal Matrix Nanocomposites
NPs: Nanoparticulates
r: Average radius of NPs (m)
TCA: Tensile Compression Asymmetry
UCS: Ultimate Compression Strength (MPa)
UTS: Ultimate Tensile Strength (MPa)
XRD: X-Ray Diffraction
$v_{p\;}$: Volume fraction of NPs (%)
